# Automotive 3.0 µm Pixel High Dynamic Range Sensor with LED Flicker Mitigation

**DOI:** 10.3390/s20051390

**Published:** 2020-03-04

**Authors:** Minseok Oh, Sergey Velichko, Scott Johnson, Michael Guidash, Hung-Chih Chang, Daniel Tekleab, Bob Gravelle, Steve Nicholes, Maheedhar Suryadevara, Dave Collins, Rick Mauritzson, Lin Lin, Shaheen Amanullah, Manuel Innocent

**Affiliations:** 1 ON Semiconductor, Santa Clara, CA 95954, USA; Minseok.Oh@microsoft.com (M.O.); Michael.Guidash@onsemi.com (M.G.); HungChih.Chang@onsemi.com (H.-C.C.); Daniel.Tekleab@onsemi.com (D.T.); Dave.Collins2@onsemi.com (D.C.); Lin.Lin@onsemi.com (L.L.); Shaheen.Amanullah@onsemi.com (S.A.); 2 ON Semiconductor, Meridian, ID 83462, USA; scott.johnson@onsemi.com (S.J.); Bob.Gravelle@onsemi.com (B.G.); Steve.Nicholes@onsemi.com (S.N.); Rick.Mauritzson@onsemi.com (R.M.); 3 ON Semiconductor, Bangalore 560025, India; Maheedhar.Suryadevara@onsemi.com; 4 ON Semiconductor, 2800 Mechelen, Belgium; Manuel.Innocent@onsemi.com

**Keywords:** image sensor, high dynamic range, automotive, LED flicker mitigation, quantum efficiency, CMOS, sensitivity, temperature dependence

## Abstract

We present and discuss parameters of a high dynamic range (HDR) image sensor with LED flicker mitigation (LFM) operating in automotive temperature range. The total SNR (SNR including dark fixed pattern noise), of the sensor is degraded by floating diffusion (FD) dark current (DC) and dark signal non-uniformity (DSNU). We present results of FD DC and DSNU reduction, to provide required SNR versus signal level at temperatures up to 120 °C. Additionally we discuss temperature dependencies of quantum efficiency (QE), sensitivity, color effects, and other pixel parameters for backside illuminated image sensors. Comparing +120 °C junction vs. room temperature, in visual range we measured a few relative percent increase, while in 940 nm band range we measured 1.46x increase in sensitivity. Measured change of sensitivity for visual bands—such as blue, green, and red colors—reflected some impact to captured image color accuracy that created slight image color tint at high temperature. The tint is, however, hard to detect visually and may be removed by auto white balancing and temperature adjusted color correction matrixes.

## 1. Introduction

Image sensors with unsurpassed high dynamic range (HDR) and light and LED flicker mitigation (LFM) performance [1,2] are powering the latest advancements in autonomous and assisting driving. These sensors employ CMOS back side illumination (BSI) technology to consistently outperform sensors of the previous generations. HDR operation along with acceptable signal-to-noise ratio (SNR) at wide temperature range is required for automotive, security, medical, IoT, factory automation, and many other applications. Linear response and high spatial resolution are preferred for accuracy of color processing and recognition rate, especially for machine vision applications in the automotive space. In order to meet these HDR LFM requirements, attenuating the number of integrated photoelectrons by temporal operation has been proposed [1]. It is efficient for high speed operation with the extension of the effective full-well capacity (FWC) by programming the duty cycle of photoelectron integration, but has a poor noise floor due to the uncorrelated double sampling (UDS) readout. Another idea using lateral overflow along with split photodiode (PD) has been presented in [3]. Using high conversion gain (HCG) with large PD and lateral overflow with small PD, low noise floor and high effective FWC are achieved. However, due to the high gain ratio between large and small PDs caused by optical gain and conversion gain ratio, dark signal non-uniformity of the small PD utilizing UDS is amplified and could be visible at elevated temperatures. This paper presents a BSI lateral overflow (LO) 3.0 µm 2.6M pixels HDR LFM CMOS image sensor with single exposure 97 dB dynamic range. In addition, the proposed sensor employs a second very short exposure subsequent to lateral overflow capture in order to extend to 120 dB dynamic range.

Much work has been done and presented on the photodiode (PD) DC and DSNU, directed to low light SNR [4,5]. For LO HDR image sensors, FD DC temporal noise has been a key consideration for the low to high light transition region SNR [6]. We have found that FD DSNU fixed pattern noise (FPN) is a dominant noise source for LO HDR pixels at high temperatures. We compare FD and PD DC and DSNU for three different wafer fabs. We then show results of using TCAD to drive process and layout changes to produce a ~28x reduction in FD DC and DSNU. All data shown is at 80 °C and an integration time of 33 msec. DC and DSNU data is normalized to the Fab1 process condition with the maximum junction electric field (Efld).

The sensor operates in the automotive temperature range from −40 to +125 °C while providing clear, colorful, and crisp images suitable for small object recognition with high probability. This work additionally studied the impact of the wide temperature range on LO HDR BSI sensor characteristics. The effects investigated included quantum efficiency (QE), sensitivity, source follower (SF) gain, pixel transaction factor (PTF) gain, color ratios, and color image quality.

## 2. High Dynamic Range Sensor with LED Flicker Mitigation

### 2.1. Pixel Description and SNR Results

**Figure 1:**
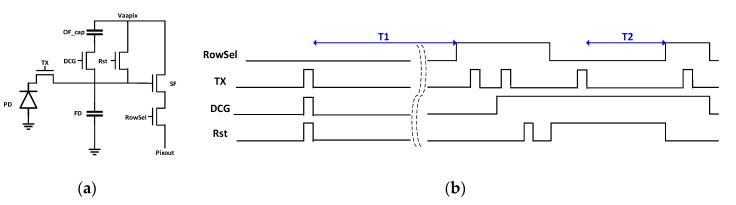
(**a**) Pixel schematic; (**b**) Timing diagram.

**Figure 2:**
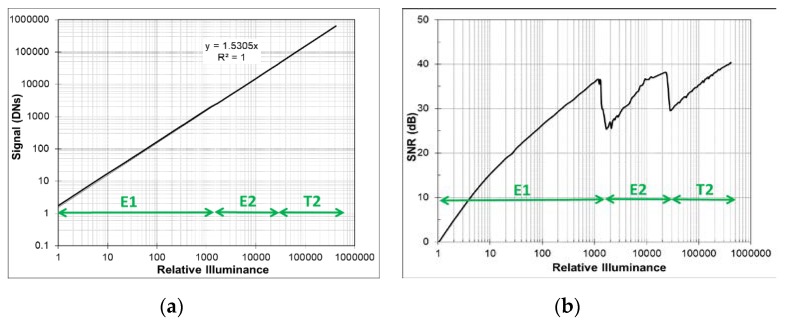
(**a**) Signal vs. illuminance plot; (**b**) Total SNR vs. illuminance plot at 100 °C (T1: 16.6 ms and T2: 0.13 ms).

Figure 1 shows the schematic of the pixel and its timing diagram. The pixel circuit consists of a PD, a transfer gate (TX), a floating diffusion (FD) to convert charge to voltage, a reset gate (Rst), a source follower amplifier (SF), a row select switch (RowSel), an overflow capacitor (OF_cap), and a dual conversion gate (DCG). Once PD is fully filled with photoelectrons under a given illumination after electronic shutter, photoelectrons overflow to FD and OF_cap, in turn. During readout after integration time of T1, overflowed photoelectrons in FD and OF_cap (E2) are sampled via UDS in low conversion gain (LCG) and photoelectrons in PD (E1) are sampled via correlated double sampling (CDS) in HCG. Combination of E1 and E2 provides 97 dB dynamic range based on single exposure. Then, normal CDS with T2 shorter than T1 in LCG is followed in order to achieve 120 dB dynamic range. As for image quality of T1 high temperature image, fixed pattern noise (FPN) needs to be considered along with temporal noise (TN) due to high contribution of temperature related FD DSNU that can be higher than TN of UDS readout in LO pixel operation. To the best our knowledge, FPN at high temperature has not been considered for LO pixel performance so far. Figure 2a shows response curve of the combination of T1 (E1 and E2) and T2 data measured at 100 °C, where T1 = 16.6 ms and T2 = 0.13 ms. Figure 2b shows the corresponding total SNR curve where

(1) $$total\;SNR=20log_{10}\frac{Signal}{\sqrt{TN^{2}+FPN^{2}}}$$

**Figure 3:**
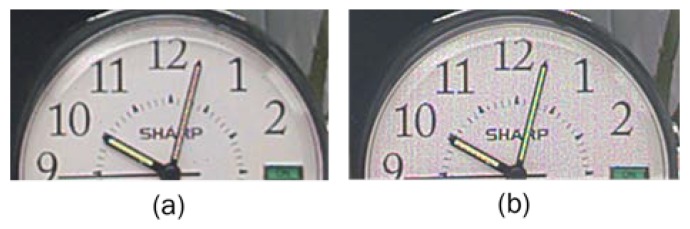
Images of E1 to E2 transition total SNR of (**a**) 32 dB, (**b**) 20 dB.

**Figure 4:**
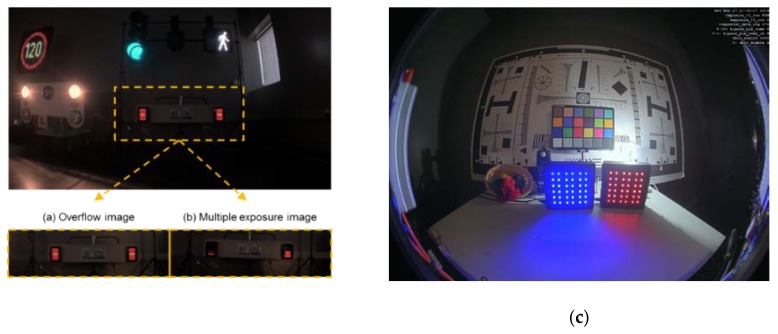
Sample HDR images in overflow (**a**), sequential (**b**), and overflow 100 °C, 33 ms tint (**c**) exposure modes.

Minimum total SNRs at both transition regions are higher than 25 dB at 100 °C. Figure 3 shows image comparison between total SNR 32 dB and 20 dB at E1 to E2 transition. It is obvious that low total SNR at transition cause poor image quality on white panel of a clock in the figure which corresponds to the transition region between E1 and E2. Figure 4 shows images of an indoor scene to mimic road conditions at night where the tail light and speed limit traffic sign flickers at 100 Hz with 10% duty cycle. Comparing images taken in overflow mode (a) and in triple exposure mode (b), it is shown that the sensor offers LFM with high image quality and resolution. Moreover, Figure 4c confirms HDR LFM image quality with no visible SNR transition noise and nice color reproduction even at 100 °C temperature and 33 ms integration time.

### 2.2. Monte Carlo Simulation for LED Detection Probability and Signal Modulation

**Figure 5:**
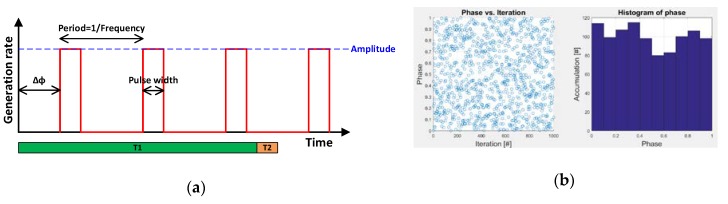
(**a**) Waveform and parameters used for Monte Carlo simulation; (**b**) An example of Δφ distribution.

Monte Carlo simulation was carried out to determine LED pulse detection probability (LDP) and LED signal fluctuation (LSF) depending on LED frequency and photoelectron generation rate (PGR) for the given pixel. Figure 5a shows waveform and parameters used for the simulation. Assuming square wave with 10% duty cycle, Δφ is a random variable with uniform probability distribution and each case is simulated with 1000 random samplings as shown in Figure 5b. Amplitude is ‘PGR/duty cycle’ where duty cycle is ‘pulse width/period’. In this simulation, T1 = 16.6 ms and T2 = 0.13 ms for 120 dB dynamic range.

**Figure 6:**
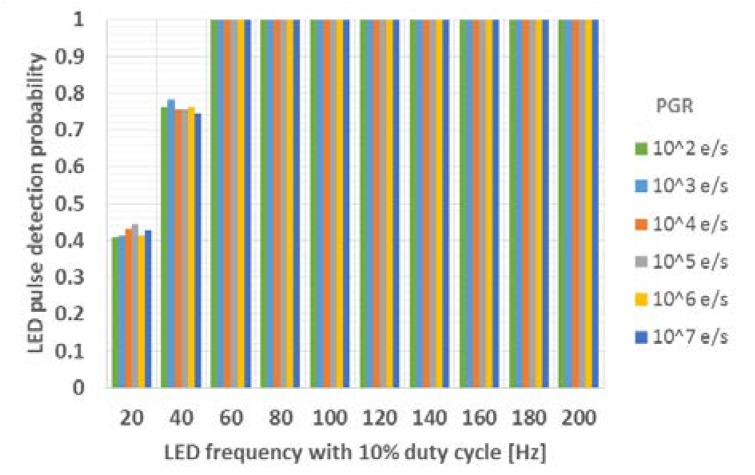
Simulation results of LED pulse detection probability vs. LED frequency vs. photoelectron generation rate.

LDP is defined as the probability to detect nothing in combined signal (T1 + T2) during integration time. As shown in Figure 6, the result of the simulation shows 100% detection probability when T1 integration time is longer than period of LED pulses.

**Figure 7:**
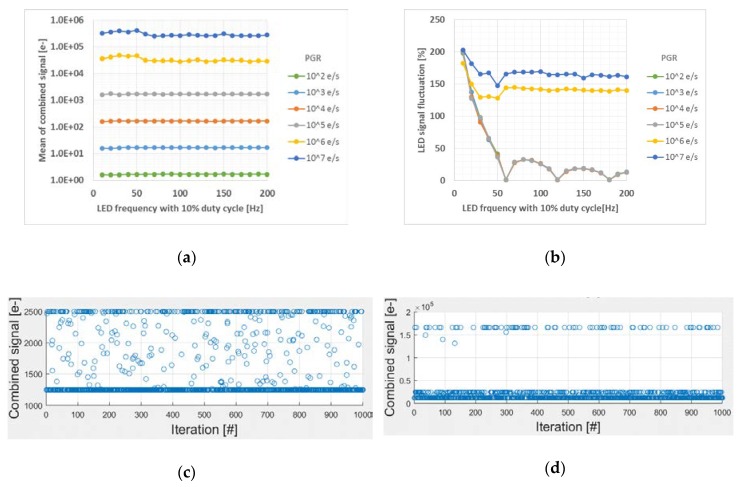
Simulation results. (**a**) The mean of combined signal, (**b**) LED signal fluctuation, (**c**) LED frequency = 80 Hz, PGR = 1 × 10^5^ e/s, and (**d**) LED frequency = 80 Hz, PGR = 1 × 10^6^ e/s

LED signal fluctuation is depicted using Figure 7. Figure 7a shows the mean of combined signal depending on LED frequency and PGR. The mean of combined signal is proportional to PGR until T2 signal participates into the combined signal (PGR < 1 × 10^6^ e-/s). As a LFM metric in this paper, LSF in percentage is introduced and defined as the standard deviation of the combined signal/the mean of combined signal×100%. As shown in Figure 7b, LSF trend before PGR reaches at 1 × 10^6^ e-/s is consistent since all the LED signal belongs to T1. In general, as LED pulse period is longer than T1 integration time, LSF is degraded due to the stochastic number of LED pulses captured during integration time as shown in Figure 7c for an example with LED frequency of 80Hz. As PGR > 1 × 10^6^ e/s, LSF becomes greater than 100% because T2 signal that captures a LED pulse stochastically starts to participate in combined signal as shown in Figure 7d.

### 2.3. Measured Sensor Parameters

The pixel is verified to provide total SNR > 25 dB at transitions up to 100 °C for automotive application. Table 1 shows the sensor performance developed in this study. As T1 integration time is longer than the period of LED pulse, the proposed pixel achieves 100% detection probability. When integrated LED signal belongs to the range of T1, LSF reaches at the theoretical limit. When integrated LED signal belongs to the range of T2, LSF degrades, but the pixel still detects LED pulse. ON Semiconductor will continue to improve the LSF along with efforts to shrink pixel size since the pixel and readout operation allow this.

## 3. FD Dark Current (DC) and Dark Signal Non-Uniformity (DSNU) Reduction for Improved SNR

### 3.1. DC and DSNU Initial Data and Analysis

**Figure 8:**
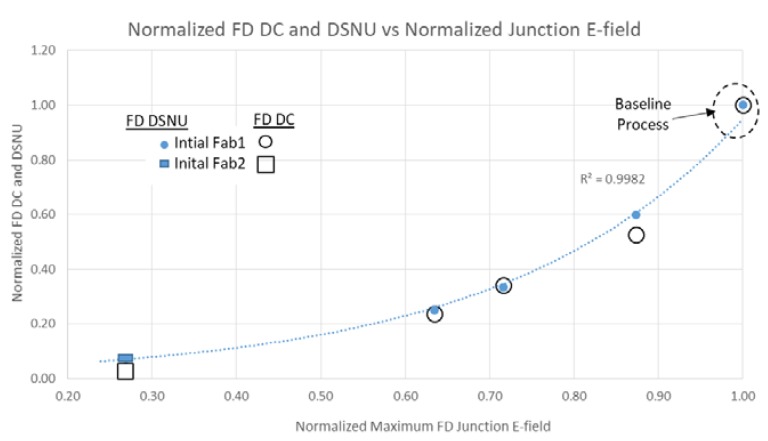
FD DC and DSNU of initial processes vs. FD junction E-field.

**Figure 9:**
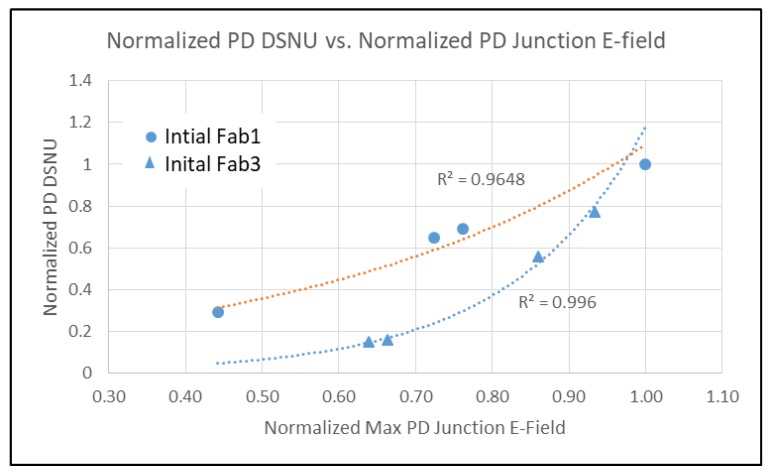
PD DSNU vs. PD junction E-field.

**Figure 10:**
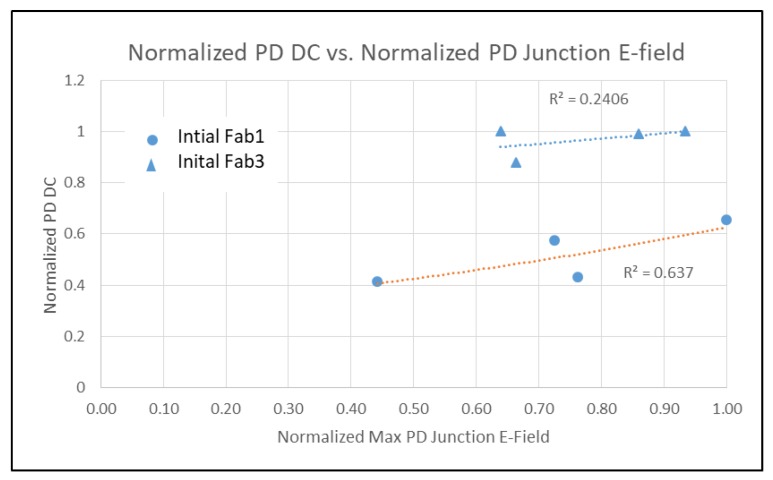
PD DC vs. PD junction E-field.

It is well known that PD DSNU is dependent on process contamination and electric field (Efld) in the PD [4,5]. FD DC and DSNU is more complicated. With LO HDR pixels, FD DC and DSNU is comprised of multiple regions, some with and without STI, and some with and without a contact. TCAD was used to determine the junction and gate Efld for the PD and FD regions for devices and various initial process conditions from three fabs [7,8]. The FD DSNU and DC vs. junction Efld is shown in Figure 8. PD DSNU and DC is shown in Figure 9 and Figure 10 respectively.

**Figure 11:**
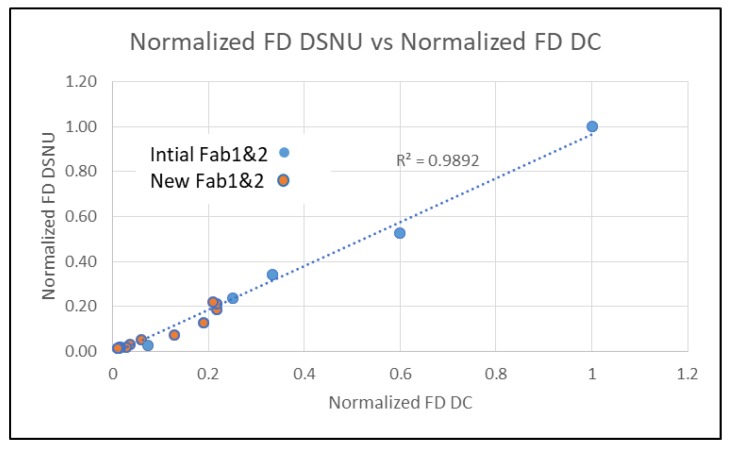
FD DC vs. FD DSNU, both initial processes and new processes.

There is an exponential relationship between DSNU and junction Efld for PD and FD. PD DC is not correlated with junction Efld, but FD DC has an exponential dependence on junction Efld. There is a linear relationship between FD DC and DSNU (Figure 11). This indicates that the generally higher FD DC and DSNU compared to the PD DC and DSNU can be reduced by decreasing the FD junction Efld.

For temperatures greater than 80 °C the FD DSNU was much larger than the FD DC shot noise, and was the limiting factor for total SNR in the transition region. A simple empirical model was determined for FD DC and DSNU as a function of gate and junction Efld based on this data and TCAD Efld results. The FD DC model was the sum of the constant junction component with an exponential dependence on junction Efld and a constant gate component with an exponential dependence on the gate to drain Efld.

### 3.2. New Process Modification Experiments for FD DC and DSNU Reduction

**Figure 12:**
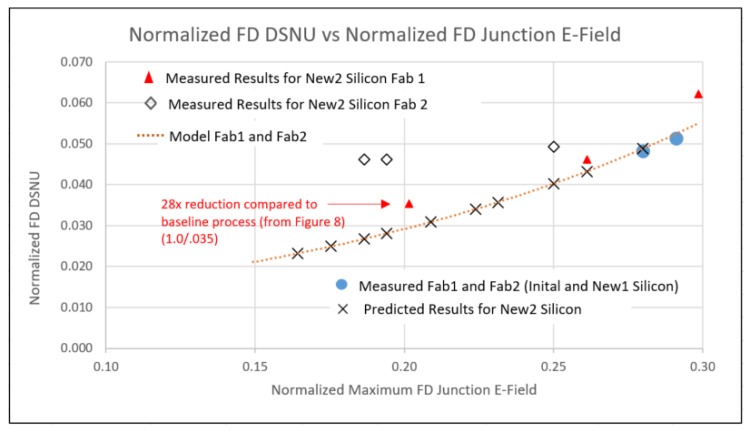
New process conditions FD DC and FD DSNU (measured vs. modeled).

**Figure 13:**
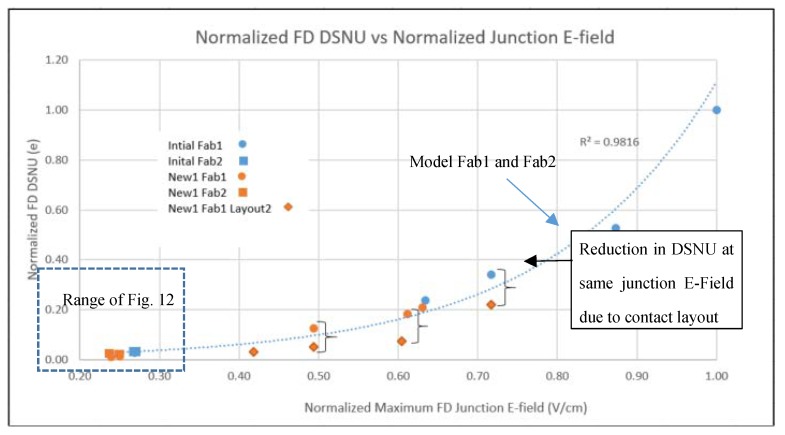
DSNU vs. FD junction E-field for new process conditions and new layout.

TCAD was used to determine a first set of process splits (New1) to reduce the FD junction and gate Eflds in two fabs. The process variables included LDD and n+ implants, STI depth, STI doping and anneals. These were fabricated and measured. Predicted results for FD DC and DSNU matched the measured data (see Figure 12 and Figure 13), still exhibiting an exponential relationship between FD DSNU and FD junction Efld. This experiment included a contact layout modification, without other modifications to the FD layout. Results indicate there was large FD DC and DSNU component related to contact placement (see Figure 13).

**Figure 14:**
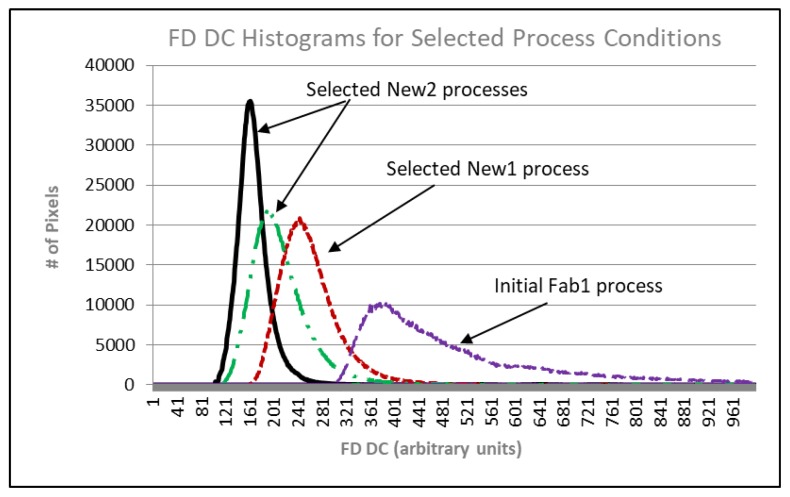
FD dark current histograms.

TCAD was next used to determine a second set of process splits (New2) to further lower FD junction and gate Eflds. These results, along with some results from the first set of process splits, and predicted results of the second process splits are shown in Figure 12. The predicted FD DSNU for Fab1 devices measured to date closely match the predicted results. An approximate 28x reduction from the initial Fab1 process condition has been demonstrated. FD DC histograms of selected Fab1 process conditions are shown in Figure 14. There is a clear reduction to the mode and tail of the histograms as the FD junction Efld is reduced.

The observed results of Fab2 devices are much higher than the predicted values. The process splits included variations for STI sidewall doping. It is suspected that the STI interface state density in Fab2 is much higher than that for Fab1 and with insufficient STI sidewall doping the DSNU floor is dominated by the STI to silicon interface. A readout timing experiment was also used to determine FD DSNU of two different FD regions; (1) a minimum geometry active region with a contact and (2) a larger active region, (~2.5× area, ~3× STI periphery, same gate periphery), without a contact. Region 1 had ~4× the DSNU of region 2. This indicates that a second dominant component related to the contact is limiting the DSNU floor for devices from Fab2.

Both FD DSNU and DC were found to have an exponential relationship with FD junction Eflds. By process modifications directed at reducing FD junction and gate Eflds a 28x reduction in FD DSNU at 80 °C and 33 msec integration time was achieved. With this level of FD DC and DSNU a total SNR > 25 dB in the transition region was achieved at 100 °C. As the FD junction and gate Eflds are reduced, FD DSNU reduction can be limited by other components (e.g., STI, contact), based on the specific fabrication process details.

## 4. Automotive Temperature Range Sensor Response Modeling and Measured Results

### 4.1. Temperature Modeling of the Pixel Behavior

**Figure 15:**
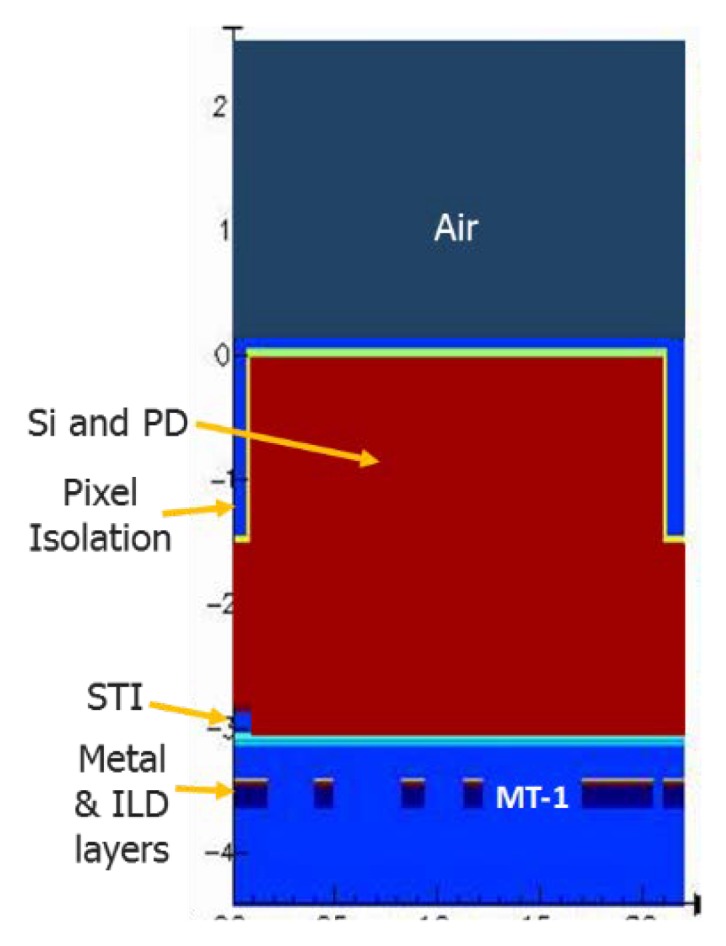
Pixel simulation model.

The Si absorption coefficient behavior across different light wavelengths has been well studied [9]. Its dependence on temperature conforms to a power law [10]. We used a 2D pixel model with no optical stack similar to Figure 15 to perform electro-optical simulations of the QE temperature dependency and consequent sensor sensitivity. The simulations used a Transfer Matrix Method (TMM) optical solver along with Si n (refractive index) and k (extinction coefficient) values obtained from [10].

**Figure 16:**
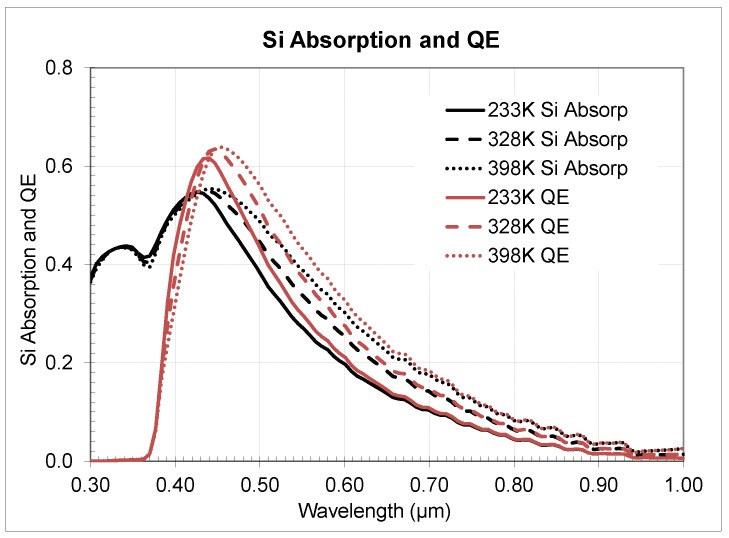
Simulated Si absorption and quantum efficiency across different temperatures for pixel without µlens, CFA, and Si ARC.

In addition, models for Si bandgap narrowing and SRH effects were included in the pixel simulations. The pixel model utilized no µlens, no CFA, and no Si anti-reflective (ARC) layers, thus the pure Si effect was studied. Simulations demonstrated that QE depends on temperature due to strong effect of temperature on the Si absorption coefficient as shown in Figure 16.

We defined image sensor sensitivity and its temperature dependence as integral product

(2) $$S(T)=\int_{350nm}^{1150nm}I_{s}(\lambda )\times T_{lens}(\lambda )\times T_{IRCF}(\lambda )\times QE(T,\lambda )\times A_{p}d\lambda$$

where *I_s_(λ)* is incoming light power, *T_lens_* is lens transmission, *T_IRCF_* is camera filter transmission, *QE(T, λ)* is pixel quantum efficiency, and *A_p_* is pixel area.

Assuming that incoming light spectra, area of the pixel, and lens and filter transmissions do not change with temperature we concluded that only *QE* defines image sensor sensitivity temperature dependence.

### 4.2. Source Follower and Pixel Transaction Factor Gain vs. Temperature

To understand the temperature impact from the shifting pixel parameters such as SF and PTF gains we performed measurements on five sensor sites across a wafer. Temperature dependency of the SF gain was studied before in consumer range from −20 to +80 °C [11], we extended the range to cover entire automotive range from −40 to +200 °C.

**Figure 17:**
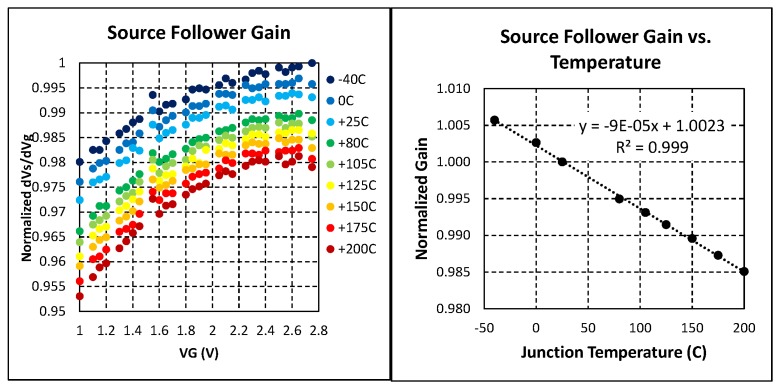
Normalized measurement results of the SF gain across automotive temperature range.

In Figure 17 we presented normalized averaged results of these SF gain measurements. When temperature increases, mobility of majority carrier decreases due to the increase of phonon scattering. As a result, transconductance and SF gain decrease accordingly. 

**Figure 18:**
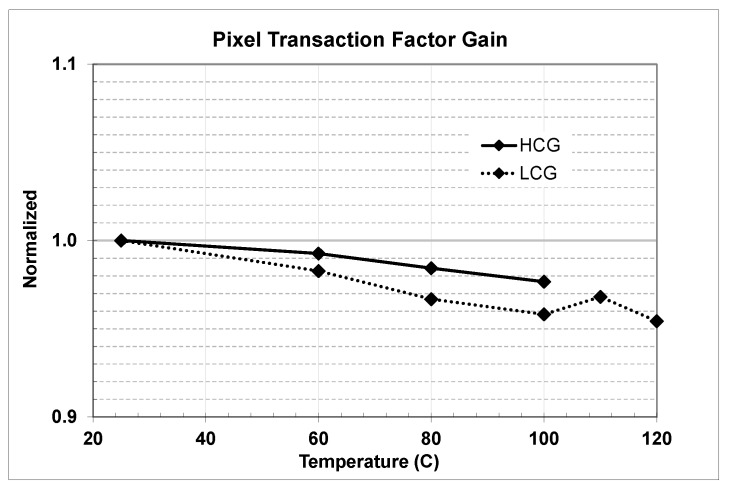
Measurement results of the pixel transaction factor gains across automotive temperature range.

Presented SF gain measurements showed reverse trend vs. temperature, earlier studies on pixel conversion and column amplifier gains showed same trend [11], then measured normalized PTF gains (DN/e) for both low (LCG) and high (HCG) conversion gains showed the similar temperature trends as depicted in Figure 18.

Measured LCG and HCG PTF gains reflected combined effect from the temperature dependencies of the pixel SF, floating diffusion, in-pixel capacitor node, and column amplifier. Measured pixel SF and PTF gains vs. temperature contributed to pixel output signal slope and were factored into further analysis

### 4.3. Quantum Efficiency, Sensitivity, and Color Ratios vs. Temperature

**Figure 19:**
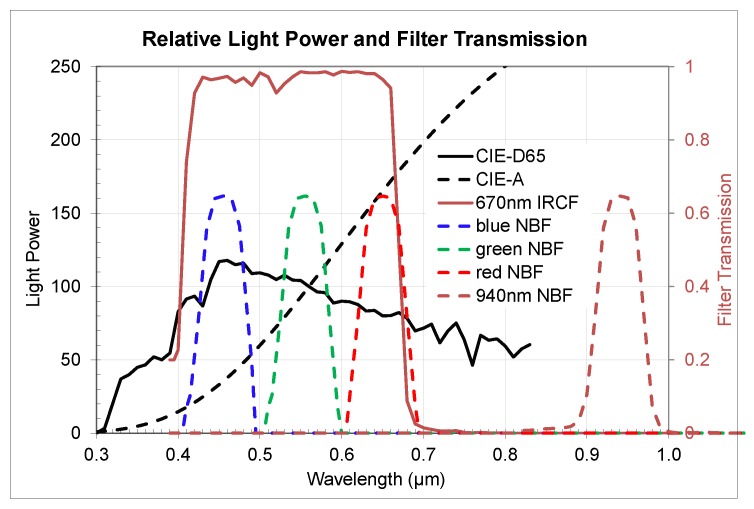
Relative light power spectral distributions and filter transmissions used for sensitivity extraction in visual and NIR ranges.

To extract sensitivity numbers for visual and near-infrared (NIR) ranges from the measured QE data we used relative light power spectral distributions and filter transmissions for both IR-cut (IRCF) and narrow band color and near-IR filters (NBFs) as shown in Figure 19.

Both CIE-D65 and CIE-A data sets are referenced in CIE publications [12].

We measured QE from 390 to 1100 nm for three samples each of the 3 µm BSI sensor presented in this work (Sensor 1) and a 2.2 µm BSI sensor (Sensor 2) using a monochromator setup with a 5 nm grating and a NIST calibrated photodiode for reference. Averaged measured QE as well as extracted sensitivity data across different temperatures was normalized to room temperature 300 K data set.

**Figure 20:**
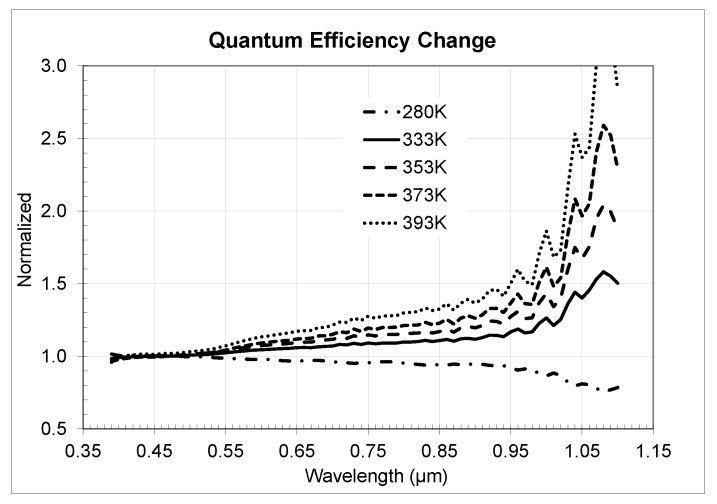
Measured normalized quantum efficiency change across different temperatures for 3 µm BSI Sensor 1.

**Figure 21:**
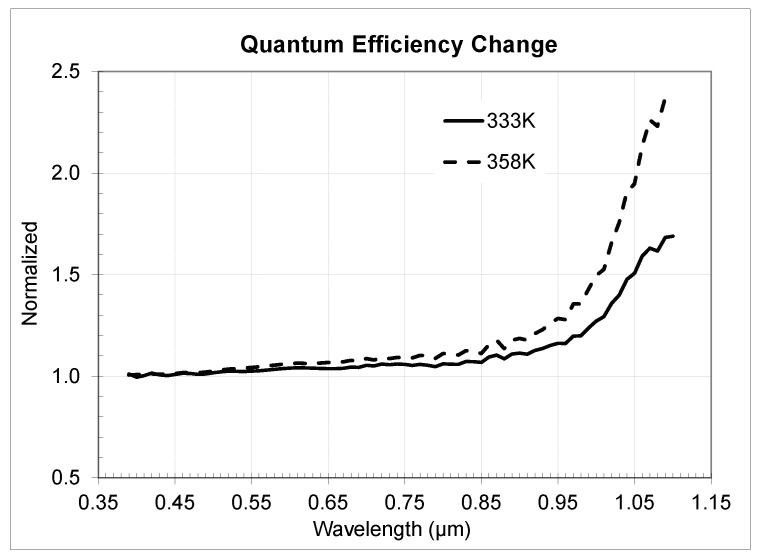
Measured normalized quantum efficiency change across different temperatures for 2.2 µm BSI Sensor 2.

In Figure 20 we presented the QE change for the 3 µm sensor and in Figure 21 for the 2.2 µm sensor correspondingly. Analysis of the data showed close matching of the observed *QE* behavior for both sensors with some very small differences probably attributable to optical stack and Si thickness variances. Temperature QE change across the wavelength follows a power law with biggest change in near-IR and relatively small change in visual range.

**Figure 22:**
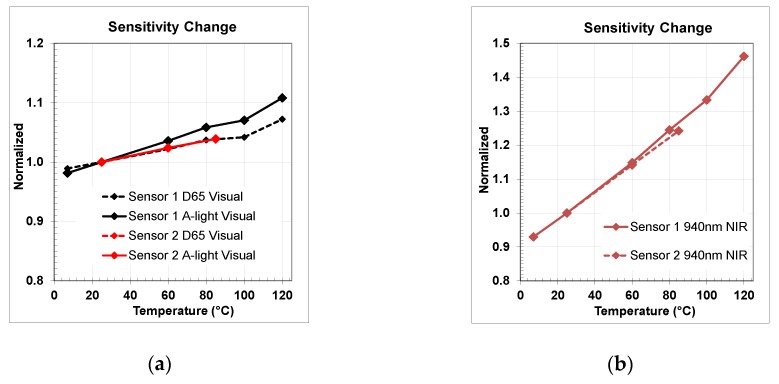
Normalized sensitivity change in visual (**a**) and NIR (**b**) ranges for both sensors.

Applying Equation (2) to D65, A-light, and measured QE data we extracted both wide band 670 nm IRCF visual and 940 nm NBF NIR normalized sensitivities and presented them in Figure 22. Sensitivity differences in the visual range were only a few relative percent comparing high temperature vs. room temperature. There was slight difference in D65 and A-light sensitivity behavior one sensor vs. another, attributable, probably, to the optical stack variances. In the NIR range, we observed relatively similar behavior for both sensors and a significant increase up to 1.46x for 940 nm band at +120 °C.

Applying Equation (2) to D65, A-light, and measured QE data we extracted both wide band 670 nm IRCF visual and 940 nm NBF NIR normalized sensitivities and presented them in Figure 22. Sensitivity differences in the visual range were only a few relative percent comparing high temperature vs. room temperature. There was slight difference in D65 and A-light sensitivity behavior one sensor vs. another, attributable, probably, to the optical stack variances. In the NIR range, we observed relatively similar behavior for both sensors and a significant increase up to 1.46x for 940 nm band at +120 °C.

**Figure 23:**
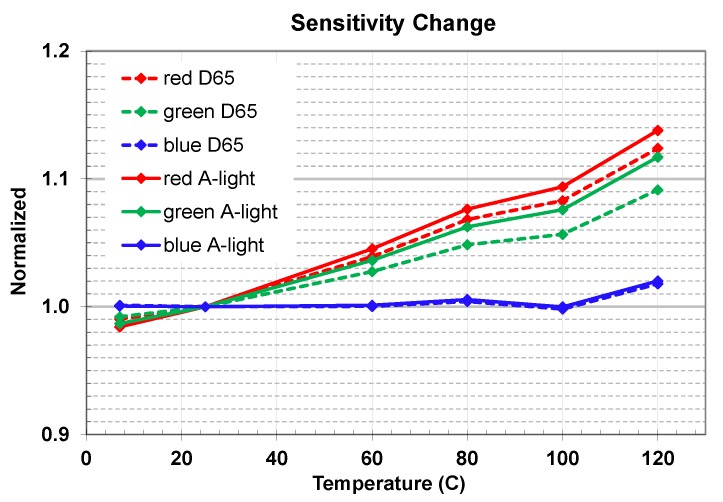
Normalized color sensitivity change vs. temperature.

Applying Equation (2) with different filters (blue, green, and red bands) to measured QE data set, we studied color sensitivity changes. Figure 23 presents normalized color sensitivities for D65 and A-light spectra correspondingly. Based on QE change vs. temperature, blue color band sensitivity change was minimal, red color sensitivity change was largest, and green color band sensitivity change was in between. All color sensitivity changes followed a linear behavior vs. temperature with the exclusion of +100 °C temperature point. We need more studies to understand the impact of the water boiling temperature point onto QE and sensitivity. Some small difference of the change in relation to the incoming light spectra D65 vs. A-light was observed, attributable to better absorption of longer wavelength photons vs. temperature, that also impacted red/green (R/G) and blue/green (B/G) color ratios.

**Figure 24:**
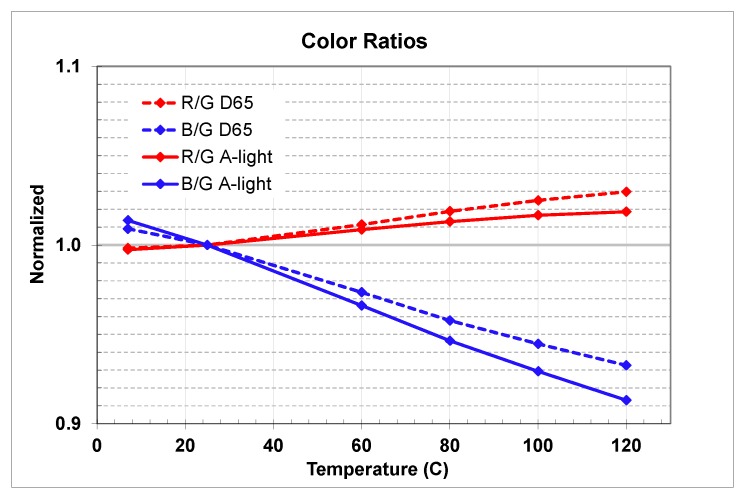
D65 and A-light R/G and B/G color ratio change vs. temperature.

In Figure 24 we presented R/G and B/G color ratios impact vs. temperature for both D65 and A-light sources.

Wider spread of the R/G and B/G color ratios at high temperatures may impact sensor color image accuracy. More spread would result in some color tinting. To validate the color accuracy impact we captured Macbeth chart images at room and high temperature equivalent to +120 °C junction. 

**Figure 25:**
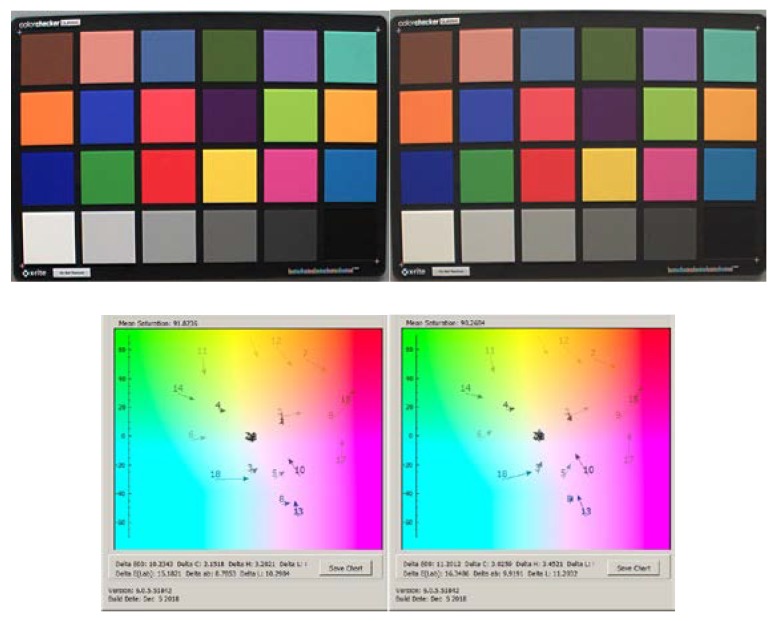
Macbeth chart images and their corresponding color accuracy at room (left) and 120 °C junction (right) temperatures.

In Figure 25, we presented Macbeth chart room temperature image capture at left and high temperature image capture at right.

Measured above change of the blue, green, and red color band sensitivity reflected some impact to captured image color accuracy, e.g., the Macbeth chart *dE* = 10.2 at room temperature vs. *dE* = 11.2 at 120 °C temperature. High temperature impact on Macbeth chart color accuracy was not large and created slight image color tint that was hard to detect visually on the captured image.

## 5. Conclusions

We presented and discussed parameters of the 3.0 µm 2.6M pixels HDR LFM image sensor effectively operating in automotive temperature range. The sensor provided total SNR in transitions greater than 25 dB up to 100 °C and 97 dB dynamic range in single and 120 dB in two exposure captures. We presented results of FD DC and DSNU 28× reduction, to provide required SNR versus signal level at temperatures up to 120 °C. We discussed temperature dependencies of QE, sensitivity, color effects, and other pixel parameters for the sensor. Comparing +120 °C junction vs. room temperature, in visual range we measured few relative percent increase while in 940 nm band range we measured 1.46× increase in sensitivity. Measured pixel source follower and transaction factor gains vs. temperature contributed to pixel output signal slope. Measured impact of sensitivity for visual bands—such as blue, green, and red colors—reflected some impact to captured image color accuracy that created slight image color tint at high temperature. The tint is, however, hard to detect visually and may be completely removed by auto white balancing and temperature adjusted color correction matrixes.

## Figures and Tables

**Table 1:** Sensor parameters

Parameter	Value
Power supply	2.8 V/1.8 V/1.2 V
Process technology	65 nm 2P5M CMOS BSI
Optical format	1/2.7 inch
Chip size	8.8^H^ mm × 8.0^V^ mm
Pixel size	3.0^H^ µm × 3.0^V^ µm
Number of active pixels	2048 × 1280 = 2.6M
Responsivity (green, D65, 670 nm IRCF, 0.9 lens transmission)	30.4 Ke–/lux·sec
Dark temporal noise (input referred noise)	270 µV_rms_
Min. total SNR at E1/E2 transition (T1 = 16.6 ms)	32 dB @25 °C, 31 dB @60 °C, 29 dB @80 °C, 25 dB @100 °C
Pixel linear full-well Dynamic range (includes TN and FPN)	140 Ke– T1: 97 dB, T1+T2: 120 dB (T1/T2 = 126)

## References

[1] Silsby C., Velichko S., Johnson S., Lim Y.P., Mentzer R., Beck J. A 1.2MP 1/3 CMOS Image Sensor with Light Flicker Mitigation. Proceedings of the IISW 2015.

[2] Velichko S., Johnson S., Pates D., Silsby C., Hoekstra C., Mentzer R., Beck J. 140dB Dynamic Range Sub-electron Noise Floor Image Sensor. Proceedings of the IISW 2017.

[3] Iida S., Sakano Y., Asatsuma T., Takami M., Yoshiba I., Ohba N., Mizuno H., Oka T., Yamaguchi K., Suzuki A. A 0.68e-rms random-noise 121dB dynamic-range sub-pixel architecture CMOS image sensor with LED flicker mitigation. Proceedings of the 2018 IEEE International Electron Devices Meeting (IEDM).

[4] Yu J., Collins D.J., Yasan A., Bae S., Ramaswami S. Hot Pixel reduction in CMOS image Sensor Pixels. Proceedings of the IS&T/SPIE Electronic Imaging.

[5] Takahashi S., Huang Y.-M., Sze J.-J., Wu T.-T., Guo F.-S., Hsu W.-C., Tseng T.-H., Liao K., Kuo C.-C., Chen T.-H. (2017). A 45nm Stacked CMOS Image Sensor Process Technology for Submicron Pixel. Sensors.

[6] Akahane N., Adachi S., Mizobuchi K., Sugawa S. (2009). Optimum Design of Conversion Gain and Full Well Capacity in CMOS Image Sensors with Lateral Overflow Integration Capacitor. IEEE Trans. Electron Devices.

[7] Schenk A. (1992). A Model for the Field and Temperature Dependence of Shockley-Read-Hall Lifetimes in Silicon. Solid-State Electron..

[8] Synopsys Sentaurus, Synopsys, Inc. https://www.synopsys.com/silicon/tcad/device-simulation/sentaurus-device.html.

[9] Green M., Keevers M. (1995). Optical properties of intrinsic silicon at 300 K. Prog. Photovolt. Res. Appl..

[10] Green M. (2008). Self-consistent optical parameters of intrinsic silicon at 300 K including temperature coefficients. Sol. Energy Mater. Sol. Cells.

[11] Xie S., Theuwissen A. (2019). Compensation for Process and Temperature Dependency in a CMOS Image Sensor. Sensors.

[12] CIE-D65 and CIE-A Data Sets. http://www.cie.co.at/.

